# MEPDB: Database of microExons in plants

**DOI:** 10.1111/nph.70456

**Published:** 2025-08-16

**Authors:** Yakub Islamov, Huihui Yu, Hongfeng Yu, Chi Zhang

**Affiliations:** ^1^ Department of Biochemistry University of Nebraska Lincoln NE 68588 USA; ^2^ Germplasm Bank of Wild Species & Yunnan Key Laboratory of Crop Wild Relatives Omics Kunming Institute of Botany, Chinese Academy of Sciences Kunming Yunnan 650201 China; ^3^ School of Computing University of Nebraska Lincoln NE 68583 USA; ^4^ School of Biological Sciences, Center for Plant Science Innovation University of Nebraska Lincoln NE 68588 USA

**Keywords:** gene structure annotation, microexon prediction, microexons, microexon‐tags, plant genomes

## Abstract

Microexons, ≤ 51‐nucleotide (nt), particularly ultra‐short ones (1–15 nt), are challenging to identify due to their small size and frequent absence in genome annotations, which limits understanding of their biological roles. Using our developed pipeline, we identified 2398 small internal microexons across 10 diverse plant species, and most of them were not annotated in the reference genomes. These microexons are grouped into 45 conserved microexon clusters based on microexon‐tags, which include the coding microexons and portions of flanking exon sequences. Leveraging these clusters, we developed a predictive method that identified 20 224 microexons across 132 plant genomes. For these microexons, we developed the Database of MicroExons in Plants (MEPDB), which includes genomic coordinates, sequences (nucleotide/amino acid), gene annotations, and updated CDS information for microexon annotations, as well as an integrated tool for RNA‐seq‐independent microexon prediction, thereby offering a comprehensive resource for plant microexon research.

**Table:** 

	Contents	
	Summary	32
I.	Introduction	32
II.	Data collection	33
III.	Database content	35
IV.	WEB interface	35
V.	Implementation	36
	Acknowledgements	36
	References	36

## I. Introduction

Microexons are typically defined as very short exons, for example, exons that are ≤ 51 nucleotides (nt) in length (Li *et al*., 2015; Ustianenko *et al*., 2017). Microexons play vital roles in various biological processes, contributing to transcript diversity, protein structure, and regulatory complexity. The biological importance of microexons has been well documented in animals, where they are known to impact protein binding domains, influence developmental processes, and facilitate adaptation to environmental stimuli. For instance, a neural‐regulated 6‐nt microexon in the nuclear adaptor Abpp1 enhanced its interaction with Kat5, and misregulated microexons in brain tissues are associated with autism spectrum disorder (Irimia *et al*., 2014; Li *et al*., 2015). In insects, alternative splicing of microexons creates multiple forms of the cell adhesion molecule fasciclin I, and amino acids inserted by alternative microexon splicing may alter the binding specificity of fasciclin I (McAllister *et al*., 1992). Emerging evidence suggests that microexons appear in plants and may similarly contribute to essential functions, including stress responses and developmental regulation. For example, a 9‐nt microexon in the Apetala 2 (AP2) domain in Arabidopsis (Ma *et al*., 2013), a 9‐nt microexon in invertase mRNAs in potato (Simpson *et al*., 2000), a 1‐nt microexon in *APC11* (anaphase‐promoting complex subunit 11) gene in Arabidopsis (Guo & Liu, 2015), and a 9‐nt microexon in the sucrose fructosyltransferase gene in wheat (Yoshida *et al*., 2004) were reported as being related to stress response and functional regulation. However, a comprehensive understanding of their roles in plant biology is hindered by the scarcity of annotated microexons in plant genomes.

Although some microexons were discovered in many eukaryotes, including vertebrates (Cooper & Ordahl, 1985; Irimia *et al*., 2014; Li *et al*., 2015; Parada *et al*., 2021), insects (McAllister *et al*., 1992; Chang *et al*., 2020), and plants (Guo & Liu, 2015; Song *et al*., 2019; Wang *et al*., 2019), many microexons remain unannotated in plant genomes, limiting our understanding of their roles in plant biology, despite their significance. The identification of microexons has distinct obstacles owing to their diminutive size; particularly when the length of a microexon is < 15 nucleotides, identification can become arduous. Conventional gene prediction methods frequently overlook these brief sequences, which may be misidentified as sequencing errors or noise, resulting in their underrepresentation in genome annotations. Unannotated microexons result in erroneous gene and protein annotations, posing a challenge for biological research (Yu *et al*., 2022).

Consequently, more sophisticated methods are required to enhance the detection of microexons in plant genomes. To address this challenge, we established computational pipelines to identify and predict microexons in diverse plants (Yu *et al*., 2022). This study addresses these gaps by developing an approach for the systematic discovery and annotation of microexons in plant genomes. We employed this bioinformatics method on 990 available RNA‐seq datasets across 10 representative plant species. A multitude of novel microexons was identified. The incorporation of these newly identified microexons enhanced gene annotations across various plant genomes, yet the substantial volume of data renders it challenging for scientists to access.

To address significant requirements, we aggregated all microexon‐related data and developed a web‐based comprehensive library of microexons to enhance the value and use of this data for efficient and effective data mining. The Database of MicroExons in Plants, MEPDB, offers comprehensive information on 20 224 microexons and 45 conserved microexon clusters across 132 plant genomes. There are 2398 high‐confidence internal microexons from 10 representative plant species, which are highlighted with RNA‐seq support data. This database enhances the precision of genome annotations, reveals new insights into plant gene regulation, and emphasizes the evolutionary and functional importance of microexons in plant biology, facilitating better applications of gene annotations and functional genomic research. The detailed information of all the datasets, including all 20 224 microexons, is available for download.

## II. Data collection

For the short microexons, which are challenging to discover, we developed a method to identify them with RNA‐seq data (Yu *et al*., 2022). Using a total of 990 publicly accessible RNA‐seq datasets, we identified 2398 small internal microexons (1–15 nt) in 10 typical plant species. About 3% of all expressed genes contain these microexons, with the majority (57–90%) being in coding areas. However, many of these microexons have inadequate annotation, with percentages ranging from 17% in the lycophyte *S. moellendorffii* to 78% in poppy (Yu *et al*., 2022).

Most coding microexons discovered by our method were not annotated in the reference genomes of rice (81%) and maize (65%), whereas fewer were missed in the Arabidopsis (27%) and soybean (23%) genome annotations (Yu *et al*., 2022). Misannotation of microexons in plant genomes often results in incorrect gene models and protein sequence inferences. If an unannotated coding microexon is located within introns of existing gene annotations, the misannotation of the microexon results in truncated protein sequences and long 5′ or 3′ untranslated regions (UTRs) due to frameshifts in the gene model and the usage of an early stop codon, or deletion of a few amino acids in the protein sequence due to microexon‐skipping in the gene model. If an unannotated microexon overlaps with or is fully located within larger exons annotated in the reference genome annotations, this microexon potentially represents alternative splicing variants or misannotation in the reference genome.

**Fig. 1:**
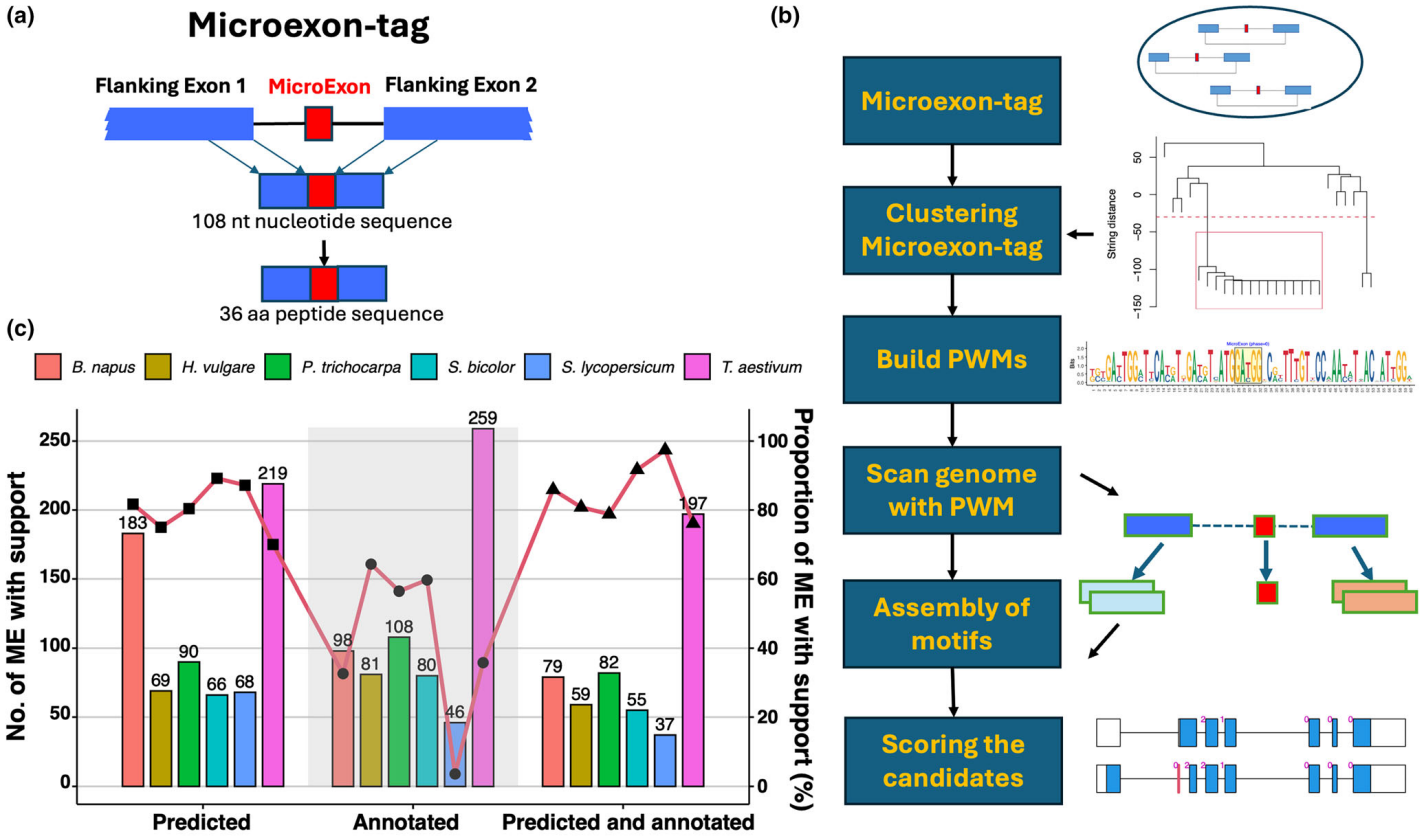
Microexon‐tag and microexon prediction method. (a) The diagram of microexon‐tag. (b) The flowchart of the microexon prediction tool. Microexons were categorized by size and phase, and within each category, they were clustered according to pairwise alignment scores of the translated amino acid sequences. A Position Weight Matrix (PWM) for nucleotide sequences within each microexon‐tag was computed. The entire genome sequence was analyzed to identify microexons and flanking exons within the microexon‐tags based on PWM scores. Ultimately, these elements in microexon‐tags were compiled to ascertain the positions of microexon‐tags and microexons. (c) The number of microexons that are predicted, annotated, or detected by both methods with RNA‐seq data support (bar plots, left *y*‐axis), and the proportion (%) of these microexons that have RNA‐seq data support (points and lines, right *y*‐axis) in sixspecies: rapeseed (*Brassica napus*), barley (*Hordeum vulgare*), poplar (*Populus trichocarpa*), sorghum (*Sorghum bicolor*), tomato (*Solanum lycopersicum*), and wheat (*Triticum aestivum*).

The size of microexons that we discovered is very short, from 1 to 15 nt. Therefore, the sequence of microexons cannot be used for sequence comparison for evolutionary studies. To study microexons and their related gene structures, the flanking exon sequences must be included. We defined a microexon‐tag, which includes the coding microexon and portions of flanking exon sequences (Fig. 1a). A microexon‐tag has 108 nt, yielding the corresponding translation of 36 amino acids (aa). The coding phase of microexons is naturally included in the microexon‐tag. Microexon‐tags span at least three exons: two flanking exons and one microexon, but occasionally, up to 5 exons when the flanking exons are too short. In certain instances, two microexons may be in close proximity, resulting in a microexon‐tag that encompasses both microexons. In extreme instances, the flanking exons begin with a start codon or end with a stop codon so that the total length is shorter than 108 nt. To compare microexons, that is, microexon‐tags, all microexon‐tags were grouped according to microexon sizes and phases, and microexon‐tags in each group were clustered based on pairwise alignment scores of translated amino acid sequences. Thus, in each cluster, the microexons have the same size and phase, and the microexon‐tags are highly similar in coding and peptide sequences. In other words, microexons in the same microexon‐tag cluster are conserved. A total of 45 clusters of conserved microexon‐tags were discovered. These 45 microexon clusters cover *c*. 70% of coding microexons in plants. Among these 45 microexon‐tag clusters, microexons exhibited variability in size and phase (0, 1, and 2). Typically, distinct microexon clusters are in different genes or gene families. Nevertheless, certain clusters are situated within the same gene family.

Utilizing a collection of 45 clusters of conserved microexon‐tags, we devised a methodology, called MEPmodeler (https://github.com/yuhuihui2011/MEPmodeler/tree/v1.0), to predict microexons in plant species independent of RNA‐seq data and genomic annotations. Fig. 1(b) shows the flowchart of the prediction method. The nucleotide sequences of 45 microexon‐tag clusters were analyzed against the NCBI plant EST (Expression Sequence Tag) database using Blastn, and a Position Weighted Matrix (PWM) for the nucleotide sequences in each microexon‐tag cluster was computed. Subsequently, we devised a method to predict microexons based on PWM scores and genomic sequences, accommodating the presence or lack of microexon flanking introns. Multiple sequence alignments demonstrated significant conservation in the positioning of microexons and the overall architecture of parent genes, particularly among flowering plants.

We applied the prediction method to 132 land plants and identified 20 224 microexons for 45 conserved microexon clusters. We incorporated them into the database for gene annotations and functional genomic research.

The predicted microexons were rigorously validated through multiple approaches. First, experimental validation was performed using RT‐PCR followed by Sanger sequencing of amplicons, which successfully confirmed all 10 tested microexons (≤ 10‐nt) in each of the four plant species examined (Yu *et al*., 2022). Second, we applied RNA‐seq datasets to 9 of 10 plant species that were used for training the 45 microexon clusters. When incorporating junction information for novel microexons during STAR remapping, 50% of these new microexons showed support from RNA‐seq reads (Yu *et al*., 2022).

For further validation, we selected six important plant species not included in the training set: rapeseed (*Brassica napus*), barley (*Hordeum vulgare*), poplar (*Populus trichocarpa*), sorghum (*Sorghum bicolor*), tomato (*Solanum lycopersicum*), and wheat (*Triticum aestivum*). For each species, RNA‐seq data from one tissue with three biological replicates were analyzed (Supporting Information Table S1). Using STAR, we mapped the RNA‐seq data to the reference genome, employing both the reference annotation GTF files and predicted microexon flanking junction files as guides. Microexons with RNA‐seq read support for both flanking introns were used for accuracy evaluation. Fig. 1(c) compares microexons identified by our prediction method, by the reference annotation, or by both approaches. The proportions of microexons with RNA‐seq support are shown for each category. As expected, microexons detected by both prediction and annotation methods exhibited the highest validation rate, with over 75% supported by RNA‐seq data. Notably, between 70.0% and 89.2%, with an average of 80.6%, of microexons identified by our prediction method have RNA‐seq support. Nonetheless, 3.6–64.3%, with an average of 42.1%, of microexons from the reference annotations possess RNA‐seq support. These results demonstrate that our prediction tool identifies microexons with higher accuracy than standard genome annotations.

## III. Database content

To share, retrieve, and store the complete annotation of microexons, microexon tags, and microexon clusters, we constructed the online database MEPDB. The current version of the database contains 20 224 small internal microexons (1–15 nt) from 132 plant genomes, and among them, 2398 from 10 representative plant genomes are highlighted for their high confidence with evidence from RNA‐seq data. All the microexons are grouped into 45 conserved microexon clusters (MEP01–MEP45), and the remaining nonconserved microexons belong to the unclassified cluster. Detailed information about each cluster is also displayed, including microexon size, microexon phase, exon block sizes in the microexon tag, position of microexon in the microexon tag, the conserved nucleotide and amino acid sequence of the microexon tag, the sequence logo, and alignment of exons' nucleotide sequences in 10 representative plant species. Each microexon has its coordinates on the reference genome, size, phase, sequence (very short), microexon tag nucleotide sequence (108‐nt), and corresponding amino acid sequence (typically 36‐aa). Other related information for each microexon includes species name, gene ID, description ID, Pfam motif IDs, motif E‐value, motif coordinates of the microexon tag sequence, CDS sequence of the full transcript, and protein sequences for the full transcript. If a microexon is not annotated in the reference genome, a new transcript ID will be created for the new transcript with the given microexon. Usually, the new transcript ID is created by adding an ‘x’ to the transcript ID of the closest annotated transcript. Both transcript IDs are displayed under the same microexon page. If a microexon can be predicted by our prediction tool, the prediction output has the prediction score and the percentage of overlap between the closest annotated transcript and the microexon tag, which is calculated by the function of *pctOverlap* in the R package, Ballgown.

## IV. WEB interface

### 1. Browsing microexon clusters and plant species

**Fig. 2:**
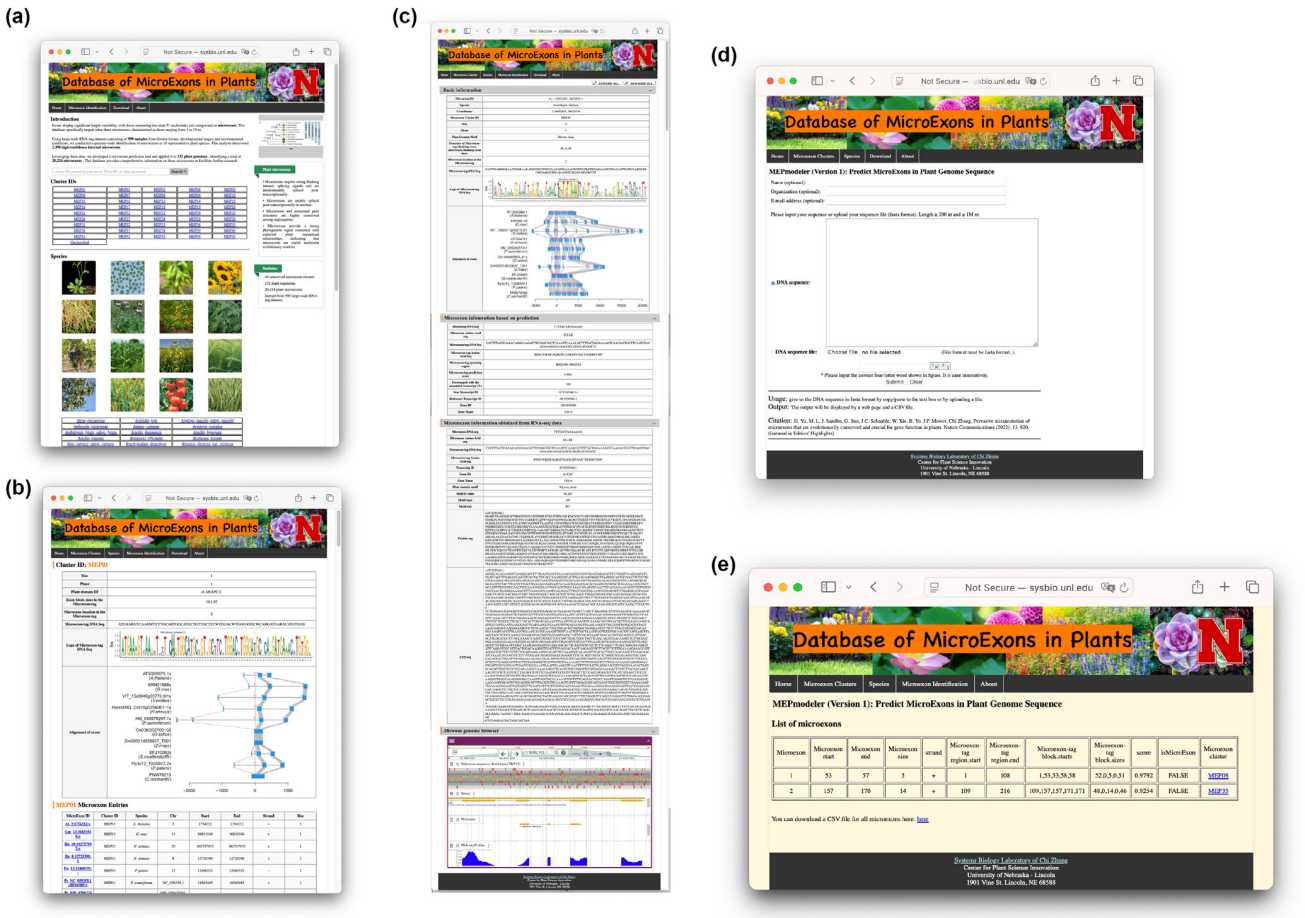
The web interface of the database. (a) The homepage of the database. (b) The webpage for one microexon cluster. (c) The webpage for a microexon. (d) The webpage for the microexon prediction tool. (e) The webpage for the result of the prediction.

The database system provides interactive access to all discovered microexons, and users may connect to the database using a web browser. Fig. 2(a) shows a screen snapshot of the front page of the database. On this page, the names of all 45 microexon clusters are enumerated, and 132 plant species are listed as well. Sixteen plant species having JBrowse are shown with species images on the front page, and 10 of them have the RNA‐seq data track in JBrowse. This page provides the main entrance for users to browse the database. Upon selecting a cluster, the corresponding page pertaining to that cluster will be displayed, as illustrated in Fig. 2(b). This new page presents comprehensive information about the cluster, with all microexons inside the cluster enumerated following the fundamental details for user selection. Upon selection of a plant species, a new page will be generated to display all microexons associated with the specified species.

### 2. Displaying the details of microexons

For a specific microexon, the information about this microexon and its microexon‐tag is displayed, such as microexon ID, coordinates in the reference genome, microexon size, phase, microexon‐tag nucleotide sequence, and so on. Additional information on the microexon is displayed as well. These types of information were obtained from: (1) reference genome annotation, if available; (2) RNA‐seq data evidence, if available; and (3) MEPmodeler prediction. A microexon may have information from one, two, or all types of resources. The microexon and its parent genes are displayed with JBrowse (Diesh *et al*., 2023).

### 3. Microexon prediction server

An online version of the microexon prediction tool was incorporated into the database, as illustrated in Fig. 2(d). A user may either paste a fragment of genomic sequence into the text field or upload a sequence file in Fasta format. The requirement of the sequence length is between 200 nt and 1 M nt. The microexon candidates will be returned and presented with microexon locations in the input sequence, coordinates of the microexon tag, and prediction scores; see Fig. 2(e) for an example. A CSV file can be downloaded with additional information regarding these microexon candidates, such as nucleotide and peptide sequences of microexon‐tags.

### 4. Microexon data for downloading

A dedicated batch download webpage is provided, offering access to the complete datasets, including the full list of microexon clusters, basic information for all 20 224 microexons, and detailed annotation, prediction, and RNA‐seq data. These different types of information about microexons are formatted for easy integration with organism‐specific genome databases and annotation pipelines. All data are also available at Zenodo (Islamov *et al*., 2025).

## V. Implementation

The database was constructed with the LAMP (Linux, Apache, MySQL, and PHP) platform. The user interface additionally accepts parameters via a URL for direct searching. This feature facilitates a link to the database from external sites, allowing users to bookmark and cite specific results directly.

## Competing interests

None declared.

## Author contributions

Huihui Yu and CZ initialized this project and curated all data. YI, CZ and Hongfeng Yu constructed the database and web pages. Huihui Yu and CZ conducted experiments for validation. CZ, Huihui Yu, and Hongfeng Yu supervised this project and drafted the manuscript. Huihui Yu and YI contributed equally to this work.

## Disclaimer

The New Phytologist Foundation remains neutral with regard to jurisdictional claims in maps and in any institutional affiliations.

## Supporting information


**Table S1** Accession numbers of RNA‐seq datasets analyzed in this study.
Please note: Wiley is not responsible for the content or functionality of any Supporting Information supplied by the authors. Any queries (other than missing material) should be directed to the *New Phytologist* Central Office.

## Data Availability

The database is freely available to all users without restriction at http://sysbio.unl.edu/MEPDB and all data saved in the database are available on Zenodo (Islamov *et al*., 2025), https://doi.org/10.5281/zenodo.15660892. The MEPmodeler (Version 1) is available on GitHub (https://github.com/yuhuihui2011/MEPmodeler/tree/v1.0). The RNA‐seq validation data analyzed in this study were obtained from public databases, with accession numbers provided in Table S1.
